# Breast Cancer Detection and Diagnosis Using Mammographic Data: Systematic Review

**DOI:** 10.2196/14464

**Published:** 2019-07-26

**Authors:** Syed Jamal Safdar Gardezi, Ahmed Elazab, Baiying Lei, Tianfu Wang

**Affiliations:** 1 National-Regional Key Technology Engineering Laboratory for Medical Ultrasound, Guangdong Key Laboratory for Biomedical Measurements and Ultrasound Imaging School of Biomedical Engineering, Health Science Center, Shenzhen University Shenzhen China

**Keywords:** breast cancer, lesion classification, malignant tumor, machine learning, convolutional neural networks, deep learning

## Abstract

**Background:**

Machine learning (ML) has become a vital part of medical imaging research. ML methods have evolved over the years from manual seeded inputs to automatic initializations. The advancements in the field of ML have led to more intelligent and self-reliant computer-aided diagnosis (CAD) systems, as the learning ability of ML methods has been constantly improving. More and more automated methods are emerging with deep feature learning and representations. Recent advancements of ML with deeper and extensive representation approaches, commonly known as deep learning (DL) approaches, have made a very significant impact on improving the diagnostics capabilities of the CAD systems.

**Objective:**

This review aimed to survey both traditional ML and DL literature with particular application for breast cancer diagnosis. The review also provided a brief insight into some well-known DL networks.

**Methods:**

In this paper, we present an overview of ML and DL techniques with particular application for breast cancer. Specifically, we search the PubMed, Google Scholar, MEDLINE, ScienceDirect, Springer, and Web of Science databases and retrieve the studies in DL for the past 5 years that have used multiview mammogram datasets.

**Results:**

The analysis of traditional ML reveals the limited usage of the methods, whereas the DL methods have great potential for implementation in clinical analysis and improve the diagnostic capability of existing CAD systems.

**Conclusions:**

From the literature, it can be found that heterogeneous breast densities make masses more challenging to detect and classify compared with calcifications. The traditional ML methods present confined approaches limited to either particular density type or datasets. Although the DL methods show promising improvements in breast cancer diagnosis, there are still issues of data scarcity and computational cost, which have been overcome to a significant extent by applying data augmentation and improved computational power of DL algorithms.

## Introduction

Cancer is one of the leading causes of female deaths worldwide. It has caused more deaths than any other diseases such as tuberculosis or malaria. The World Health Organization (WHO) agencies for cancer research (ie, International agency for cancer research (IARC) and American Cancer Society) report that 17.1 million new cancer cases are recorded in 2018 worldwide [1]. WHO estimates that cancer incidences might increase to 27.5 million by 2040, with an estimated 16.3 million deaths expected as a result of cancer [1].

Breast cancer is among the 4 leading cancers in women worldwide (ie, lung, breast and bowel [including anus], stomach, and prostate cancers). The IARC statistics show that breast cancer accounts for 25% of all cancer cases diagnosed in women worldwide. Around 53% of these cases come from developing countries, which represent 82% of the world population [1]. It is reported that 626,700 deaths will occur only in 2018 [1]. Breast cancer is the leading cause of cancer death among women in developing countries and the second leading cause of cancer death (following lung cancer) among women in developed countries.

In breast, the cancer cells may spread to lymph nodes or even cause damage to other parts of the body such as lungs. Breast cancer more often starts from the malfunctioning of milk-producing ducts (invasive ductal carcinoma). However, it may also begin in the glandular tissues called lobules or other cells or tissues within the breast [1]. Researchers have also found that hormonal, lifestyle, and environmental changes also contribute to increasing the risk of breast cancer [2,3].

**Figure 1:**
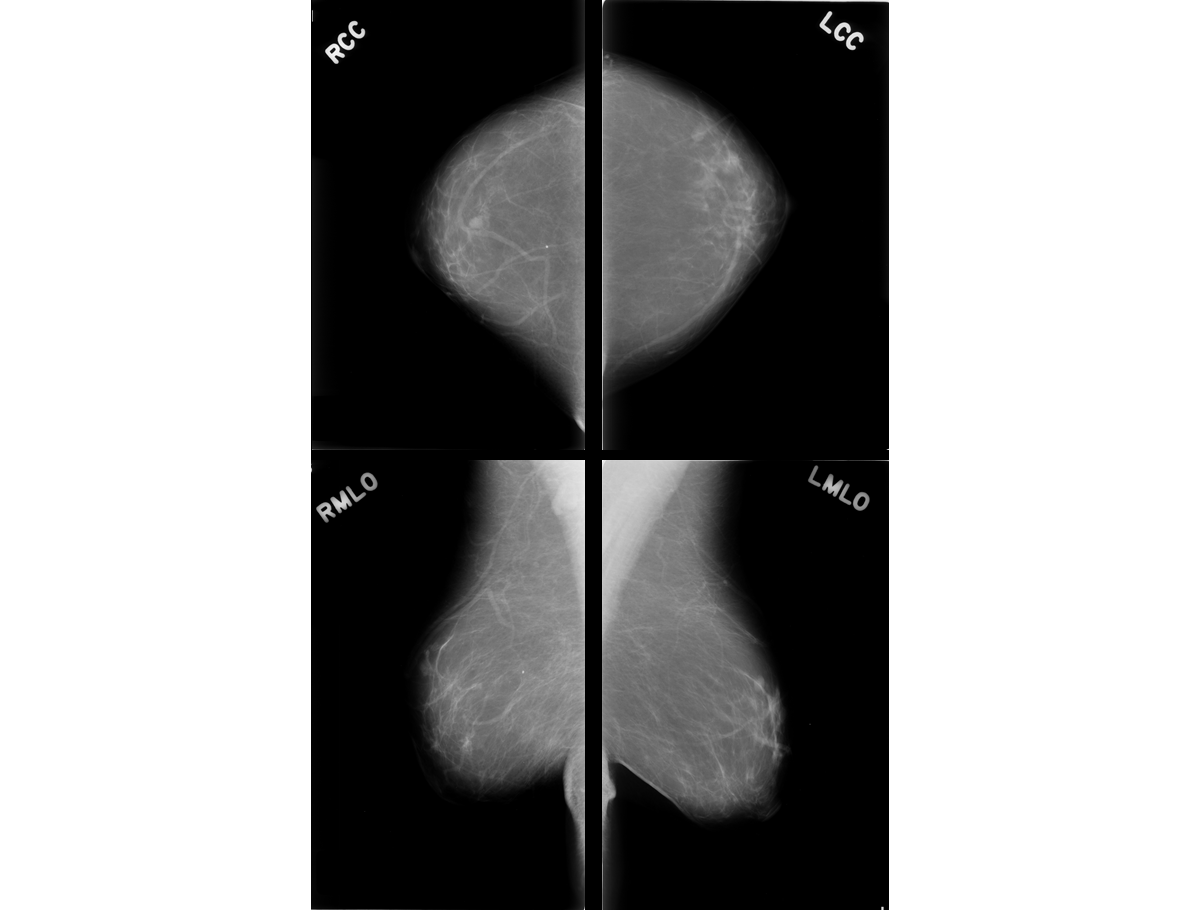
Multiview breast mammogram of a patient. The first column presents two views of the right breast: right craniocaudal (RCC) view and right mediolateral oblique (RMLO) view. The second column presents two views of the left breast: left craniocaudal (LCC) view and left mediolateral oblique (LMLO) view.

To visualize the internal breast structures, a low-dose x-ray of the breasts is performed; this procedure is known as mammography in medical terms. It is one of the most suitable techniques to detect breast cancer. Mammograms expose the breast to much lower doses of radiation compared with devices used in the past [4]. In recent years, it has proved to be one of the most reliable tools for screening and a key method for the early detection of breast cancer [5,6]. The mammograms are acquired at 2 different views for each breast: craniocaudal (CC) view and mediolateral oblique (MLO) view (Figure 1).

In this review, we present the recent work in breast cancer detection using conventional machine learning (ML) and deep learning (DL) techniques. The aim of this work was to provide the reader with an introduction to breast cancer literature and recent advancements in breast cancer diagnosis using multiview digital mammograms (DMs). The survey aimed to highlight the challenges in the application of DL for early detection of breast cancer using the multiview digital mammographic data. We present the recent studies that have addressed these challenges and finally provide some insights and discussions on the current open problems. This review is divided into 2 major parts. The first part presents a brief introduction of different steps of a conventional ML method (ie, enhancement, feature extraction, segmentation, and classification), whereas the second part focuses on DL techniques, with an emphasis on multiview (ie, CC and MLO) mammographic data. The present DL literature can be characterized for breast density discrimination, detection, and classification of the lesion in breast cancer in the multiview digital mammographic data. The rest of this review is organized as follows.

## Methods

### Conventional Machine Learning Pipeline

**Figure 2:**
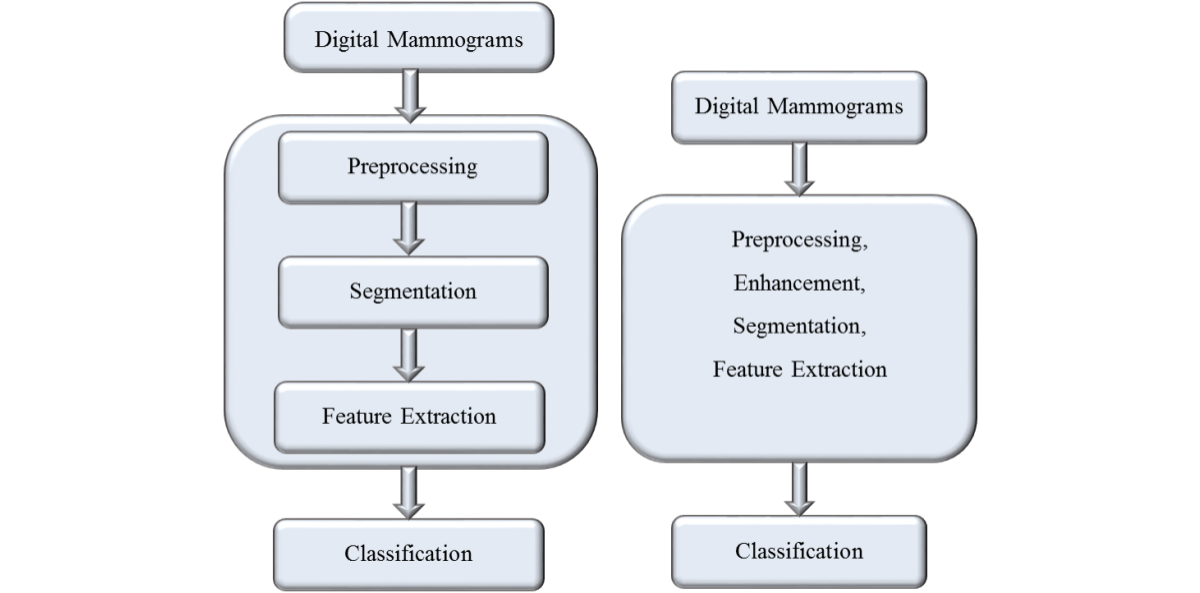
Difference between 2 pipelines: conventional machine learning pipeline (left) and deep learning pipeline (right).

In this section, we present various steps involved in a computer-aided diagnosis (CAD) system using the conventional workflow. The steps involved are outlined in Figure 2 and are discussed briefly as follows.

### Breast Profiling and Preprocessing

**Figure 3:**
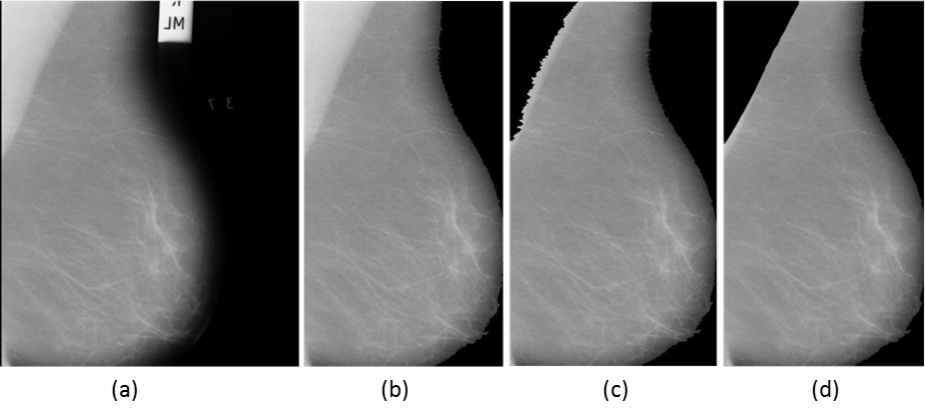
(a) Original mammogram image 1024×1024. (b) Preprocessing to remove annotations. (c) pectoral muscle (PM) removal by region growing. (d) PM removal by adaptive segmentation.

Mammogram preprocessing is one of the primary steps in a CAD system. In the preprocessing step, the unwanted objects are removed from the mammograms, which include annotations, labels, and background noises as can be seen in Figure 3. The preprocessing helps the localization of region for abnormality search. In mammogram preprocessing, one of the major challenges is to accurately define the pectoral muscle (PM) boundary from the rest of the breast region. The PMs are mostly present in MLO views of the mammograms. The presence of PMs in the MLO view can interrupt the automatic detection of lesions and can increase the false positive (FP) alarms. Many studies advocated the removal of PMs [7-15] for improving the diagnostic accuracy of the CAD system. Thus, successful removal of PMs is vital to avoid false detection. Moreover, it also reduces the time complexity and improves the accuracy apart from avoiding the intra-observation discrepancies.

### Image Enhancement Techniques

Image enhancement techniques are used to improve the mammogram’s quality in terms of improving the contrast and enhancing its readability. It helps the system to detect the mammographic lesions with poor visibility and contrast by improving it. The major goal of mammogram enhancement is to improve the image quality on the mammograms with low contrast. The low-contrast regions with small abnormalities are often concealed in surrounding tissues, leading to a misdiagnosis. The image enhancements improve the overall quality of the images, thus making it relatively easier for the reader and CAD systems to detect these subtle abnormalities. The enhancements may add distortions to the anatomical characteristics of an image or amplify the noises. Thus, only those methods would be acceptable that maintain a similar appearance to the original image. Recently, with the introduction of direct digital technology in mammography, with dynamic range, improved contrast, and signal to noise ratio, there is a limited scope of mammogram enhancement.

The enhancement techniques are generally divided into 3 categories: spatial domain, frequency domain, and a combination of spatial and frequency domain techniques [16]. However, these techniques can be characterized into 4 types [17] based on their particular usage: namely, conventional, region-based, feature-based, and fuzzy enhancement techniques. The primary aim of enhancements is to improve the quality of mammograms to achieve high diagnostic performance. The conventional methods can be adapted for local as well as global enhancement of the mammograms. However, the conventional methods have a tendency to enhance the noise factor as well. On the other hand, the region-based methods are suitable for contrast enhancements of particular regions of interest (ROIs) with varying shapes and sizes. The region-based methods help to enhance the anatomical details of the ROIs without any addition of artifacts. These methods are generally well suited for microcalcification enhancements in breasts with dense tissues. The feature-based enhancement techniques are applied on mammograms with calcifications as well as masses. The multiscale transforms such as wavelets are used because of their dilation and translation properties that are best suited for nonstationary signals. The low frequencies are suppressed, whereas only higher frequencies are kept by applying a threshold. Thus, the reconstructed images only contain highest frequencies with possible lesion regions. Finally, the fuzzy enhancement technique uses the maximum fuzzy entropy principle on the normalized mammograms to enhance the contrast and suppress the noise. These techniques are effective to enhance the mass contours and present the fine details of mammogram features.

### Mammographic Mass Segmentation Techniques

The segmented region is vital for feature extraction and detection of abnormal tissues in the breast, and it needs to be well focused and precise. Therefore, the segmentation is important to extract an ROI that provides a precise measurement of breast regions with abnormalities and normal regions. Segmentation involves the fundamental step of separating the breast region from the background and aims to separate the breast regions from the other objects. It is an important step to preserve the margin characteristics of mammograms before any further processing.

**Figure 4:**
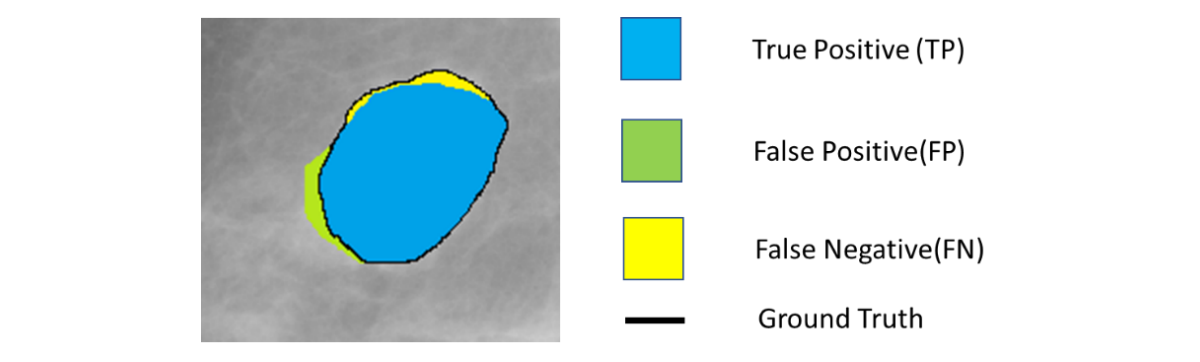
Pixel-level illustration of true positive, false positive, and false negative compared with ground truth.

The segmentation aims to extract ROIs with possible masses, and it may involve partitioning of the mammogram into several nonoverlapping regions with candidate mass lesions. At the detection stages, higher sensitivity rate and more FPs are expected. Figure 4 illustrates the FP detection at pixel level compared with ground truth boundary. These FPs can be removed after the classification stage. In the literature, many researchers have devised automatic [18-21] as well as ensemble segmentation and classification [22-24] algorithms by combining several techniques to reduce the FPs at the detection stage. In general, the segmentation techniques can be characterized as thresholding based, region based (ie, region growing and region clustering), feature and edge based. We briefly summarize the advantages and disadvantages of segmentation techniques in Table 1.

**Table 1:** Summary of advantages and disadvantages of segmentation methods.

Methods	Advantages	Disadvantages
GT^a^	Widely used as preprocessing step in image processing as these methods are easy to implement	Not suitable for segmentation of ROIs^b^, as GT methods produce high false positive detections
Local thresholding	Works well compared with GT, sometimes used to improve the GT results	Widely used in literature as initialization step of other algorithms, but local thresholding fails to separate the pixels accurately into suitable regions
Region growing	Uses pixel connectivity properties to grow iteratively and sum up the region having similar pixel properties	Need initialization point, that is, a seed point to begin with and highly dependent on initial guess
Region clustering	No seed point required to initialize; it can directly search the cluster regions.	Total number of clusters need to be predefined at initial stage
Edge detection	Highly suitable for detecting the object boundaries and contours of the suspected ROIs	Requires some information about object properties
Template matching	Needs ground truth and are easily implemented. Easy implementation; if the prototypes are suitably selected, it can produce good results.	Need prior information about the region properties of the objects such as size, shape, and area.
Multiscale technique	Do not require any prior knowledge about object properties	Requires empirical evaluation to select the appropriate wavelet transform
	Easily discriminate among the coefficients at different level and scale of decompositions	Need to select scale of decompositions

^a^GT: Global thresholding.

^b^ROI: region of interest.

### Conventional Feature Extraction Techniques

In ML methods, learning the significant or most informative features from the medical images plays a vital role, as these features are used as discriminators in later stages for segmentation or classification. Most of these features are manually designed (handcrafted) based on clinicians’ experience and prior knowledge about the target domain. Thus, the ML methods can be more problem oriented and often make it difficult for a nonexpert to exploit the full potential of the method. The feature extraction is the step that characterizes the features of a specific region. The significant features are retained for the classification step. To measure features from the ROIs, its properties such as mass size, regular or irregular shapes, homogeneity of boundaries, and density of tissues are utilized [25]. It is widely known that because of the variation in properties of normal and diseased tissues, feature space exhibits a large and complex nature. Most features are not significant when separately studied. However, when combined with other features, they can represent significant information that is helpful for the classification step. The performance of the algorithm is affected, and the complexity of the classifiers increases when excessive use of features is done. Thus, drawing the optimal features from images is very crucial. A number of feature selection techniques such as principal component analysis (PCA) [26], linear discriminant analysis (LDA) [27], filtering techniques such as chi-square test [28,29], and many other feature reduction methods [30] are used to select the most discriminative features to avoid overfitting and reduce the redundancy in feature space. On the basis of the feature characteristics, the feature space can be divided into 3 categories: morphological (shape or geometric) features, texture or statistical features, and multiresolution features.

### Classification Techniques

**Figure 5:**
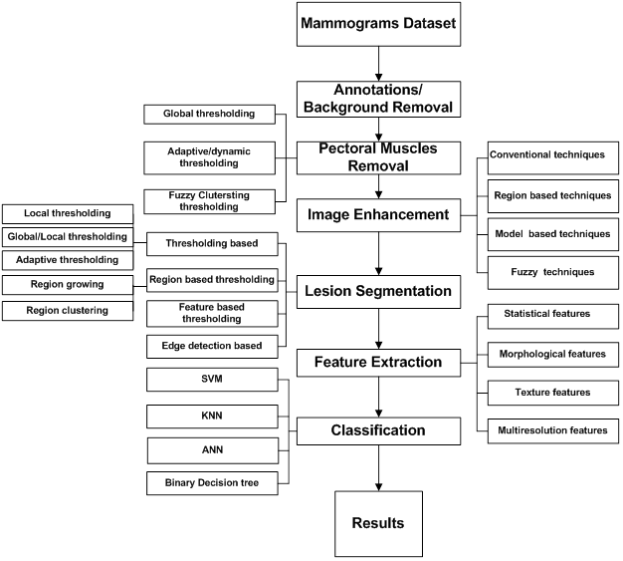
An overview of mammogram processing using computer-aided diagnosis based on machine learning algorithms.

Classification is the last step to determine the lesion under observation is normal or cancerous regions. If it is classified as a cancerous region, further classification is done to determine the pathology of cancer, ie, benign or malignant. The classification step itself is heavily dependent on other intermediate steps, especially segmentation and feature extraction. In breast cancer classification, some of the commonly used classifiers include support vector machine (SVM) [31-34], artificial neural network (ANN) [10,13,21,35,36], k-nearest neighbor (KNN) [37,38], binary decision tree [39], and simple logistic classifier [34,40]. The performance of the classifier can be improved using some feature selection method to remove the redundant features and keep only the most discriminative features. An overview of CAD system based on ML algorithms for breast cancer diagnosis using mammographic data is illustrated in Figure 5.

### Summary of Machine Learning Methods

A substantial amount of research on breast mass, microcalcification detection, and classification can be found in literature [22-24,31,34,41-45]. Masses are more challenging to detect compared with microcalcifications because the mass features may be concealed or alike to those of normal breast parenchyma. Thus, the detection of masses is still an open challenge in breast cancer detection. We also note that masses greatly vary in size [15,46], which makes it more challenging to detect. Another major limitation of the conventional ML studies is that mass analysis has not been done by defining some suitable scale for the range of masses. By defining the range of sizes, mass regions can be approached at the coarsest scale of description. However, a more confined approach is required to detect the boundaries of masses. Moreover, the variations in widths, lengths, and density spiculations that are associated with cancerous lesions and the varying scales require a more rigorous characterization and analysis. Apart from mass detection, architecture distortion and the detection of bilateral asymmetry are also important research topics in mammograms [34]. The new developments must cope and overcome with the challenges that existing algorithms exhibit by improving the performance. Furthermore, commercial CAD systems have achieved a reasonable degree of effectiveness to detect masses and calcifications. Future work on CAD systems for breast cancer diagnosis should focus on improving the performance. Feature extraction is one of the important steps in developing a CAD system. A broad variety of features for the characterization of breast cancer have been developed in the past years. Hence, more researches seem to be necessary to measure features robustness that can produce a high classification accuracy rate. Selecting the optimal feature subset for supervised learning problems requires an exhaustive search. The discriminative power of features used in CAD systems varies. Although some are highly significant for the discrimination of mammographic lesions, others are redundant or even irrelevant. Hence, automatic extraction of a subset of features from a higher dimensional feature vector is a common module in mammography CAD approaches.

### Deep Learning, an Overview

DL algorithms have made significant improvements in performance compared with other traditional ML and artificial intelligence [47]. The applications of DL have grown tremendously in various fields such as image classification [47], natural language processing [48], gaming [49]; and, in particular, it has become very popular in the medical imaging community for detection and diagnosis of diseases such as skin cancer [50,51], brain tumor detection, and segmentation [52].

The DL architectures can be characterized into 3 categories: unsupervised DL networks, also known as generative networks; supervised networks or discriminative networks; and hybrid or ensemble networks.

Convolutional neural network (CNN) is a state-of-the-art DL technique that is comprising many stacked convolutional layers [47]. The most common CNN discriminative architecture contains a convolutional layer, a maximum pooling layer to increase the field of view of the network, a rectified linear unit (ReLU), batch normalization, a softmax layer, and fully connected layers. The layers are aligned on top of each other to form a deep network that can the local and spatial information from this layer when a 2D or 3D image is presented as an input [53].

The AlexNet [47] architecture was one of the first deep networks for improving the ImageNet classification accuracy by a significant stride than the existing traditional methodologies. The architecture contained 5 convolutional layers proceeded by 3 fully connected layers. The ReLU activation function for the nonlinear part was introduced by replacing the traditional activation function such as Tanh or Sigmoid functions used in neural networks. ReLU has fast convergence as compared to sigmoid, which suffers from the vanishing gradient problem.

Later, VGG 16 architecture was proposed by visual geometry group (VGG) [54], Oxford University. The VGG improved the AlexNet architecture by changing the kernel size and introduction of multiple filters. The large kernel-sized filters are replaced (ie, 11×11 in Conv1 and 5×5 in Conv2, respectively) by multiple 3×3 kernel-sized filters that are placed one after another. The multiple smaller kernel filters improve the receptive field compared with a larger size kernel, as multiple nonlinear layers increase the depth of the network. The increased depth enables to learn more complex features at a lower cost. Although VGG achieved very good accuracy on classification tasks for the ImageNet dataset, it is computationally expensive and requires huge computational power, both in terms of storage memory and time. Thus, making it inefficient because of the large width of convolutional layers.

The GoogleNet [55] proposed the idea that most of the connection in dense architecture and their activations in the deep network are redundant or unnecessary due to correlations between them. This makes the network computationally expensive. Therefore, GoogleNet implied to have a most efficient network with sparse connections between the activations. GoogleNet introduced the inception module, which effectively computes sparse activation in a CNN with a normal dense construction. The network also uses 3 different convolutions sizes (ie, 5×5, 3×3, and 1×1) to have a better receptive field and extract details from very small levels. One of the important salient points about the inception module is that it also has a so-called bottleneck layer (1×1 conv.) that helps in massive reduction of the computation requirement. Another change that GoogleNet introduced is global average pooling at the last convolutional layer, thus averaging the channel values across the 2D feature map. This results in a reduction of the total number of parameters.

With increasing network depth, the accuracy of the network is saturated and thus degrades rapidly. This degradation is not caused by overfitting problem, but with the addition of more layers, the training error also increases that leads to degradation problem. The degradation problem was solved by introducing the residual network (ResNet) by He et al [56]. The residual module was introduced to effectively learn the training parameters in a deeper network. They introduced skip connections in convolutional layers in a blockwise manner to construct a residual module. The performance of ResNet is better than VGG and GoogleNet [57].

### Deep Learning for Breast Cancer Diagnosis

Many researchers have used DL approaches in medical image analysis. The success of DL is largely depending on the availability of large number of training samples to learn the descriptive feature mappings of the images, which give very accurate results in classification. For example, the image classification task, the network is trained over more than 1 million images with more than 1000 class data. However, in the case of medical images, the amount of available training data is not that big in size. Moreover, it is also difficult to acquire a large number of labeled images, as the annotation itself is an expensive task and for some diseases (eg, lesions) are scarce in the datasets [58]. In addition, annotation of these data samples, if exist, in different classes suffers from intraobserver variations, as the annotation is highly subjective and relies on the expert’s knowledge and experience. To overcome the data insufficiency challenge, many research groups have devised different strategies: (1) using 2D patches or 3D cubes instead of using the whole image as input [59,60], which also reduces the model parameters and alleviates overfitting; (2) by introducing data augmentation using some affine transformations (translation, rotation, and flipping [61,62]) and training the network on the augmented data; (3) by transferring learning approach using pretrained weights [63,64] and just replacing the last layers by the new targeted class instead; and (4) using trained models with small input sizes and then transforming the weights in the fully connected layers into convolutional kernels [65].

### Search Strategy for Study Selection

To select the relevant recent studies on breast cancer diagnosis, we consider the studies in the past 5 years from well-known publishing platforms such as PubMed, Google Scholar, MEDLINE, Science Direct, Springer, and Web of Science databases. The search terms convolutional neural networks, deep learning, breast cancer, mass detection, transfer learning, and multiview are combined.

## Results

### Convolutional Neural Networks for Breast Cancer Diagnosis

In this section, we first present the methods that used breast density estimation methods as a tool for early diagnosis. Second, the methods used transfer learning and image features classification of suspected lesions into mass and normal class. Finally, we present the segmentation methods using semantic features for localization of masses and classifying the pathology.

### Convolutional Neural Network for Breast Density Estimation

Mammographic density is an important indicator of early breast cancer detection. In the United States, more than 30 states have agreed to use breast density as an earlier risk marker for cancer screening programmes [66]. The qualitative assessment is highly subjective, and there are wide variations in scoring results among the radiologists [67]. Recent studies also reveal that commercial software to assess the breast score tends to give mixed results by either over or under reporting when compared with assessment by radiologists [68,69]. The DL algorithms for density assessment can significantly reduce the burden of manual scoring for the radiologist and improve the performance for risk assessment [66].

One such attempt has been made by Mohamed et al [70] using CNN to classify the DMs based on breast densities. The Breast Imaging Reporting and Data System (BI-RADS) characterizes the densities into 4 classes. The discrimination of this breast densities acts as a risk marker for breast cancer, and radiologist can visually access the results. The study is focused on distinguishing the 2 difficult categories: scattered dense and heterogeneous dense breast tissues. Their method showed promising results for classification.

In another study, Ahn et al [71] presented a CNN-based approach for breast density estimation. The CNN was trained to learn image features from the image patches extracted from the whole mammograms and classify them as fatty and dense class tissues. The local and global statistical features were used to train the CNN. Wu et al [72] presented the application of deep neural network (DNN) for classification of breast densities in DMs. The study comprised 20,000 screening mammograms labeled as 4 class breast densities (ie, fatty, fibro-glandular dense, heterogeneously dense, and extremely dense). A scratch-based CNN with dense convolutional layers was used to discriminate the breast densities in the multiview data.

In a similar study, Xu et al [73] classified the breast density estimation method using residual CNN. The method worked efficiently for both single and multiview images (ie*,* CC and MLO). Their study aimed to use the residual CNN to discriminate the BI-RADS densities into 4 categories. The residual CNN consisted of 70 layers with 7 residual learning blocks. In addition, 2 other networks with 36 and 48 weighted layers but less residual blocks were also trained to compare the performance. The ResNets could minimize the cross-entropy loss to maximize classification accuracy. Their results showed that with increased residual layer, the classification accuracies improved. However, the computational cost was increased.

Kallenberg et al [74] proposed an unsupervised DL technique to classify the breast density and risk score in the segmented regions. The method uses conventional sparse autoencoder (CSAE) for learning the features. For mammographic density score, 3 class labels were used: PMs, fatty breast tissues, and dense breast tissues. For the mammographic texture score, 2 classes were considered (ie, cancer and normal patches). This score was used as a threshold to segment that tissue from the breast. Dice score showed the goodness of segmented versus the ground truth. The CSAE model was trained and tested for 3 different datasets, and the results showed a positive relationship with the scores obtained manually by experts.

Ionescu et al [75] proposed a CNN-based density estimation method to assist the radiologist in risk scoring. The CNN is trained to assess the visual analog score from unseen images. The method showed a strong correlation and match concordance indices results when compared with 2 independent readers in a clinical environment.

Geras et al [76] in their study used deep convolutional neural network for prediction of breast densities in multiview data. The method predicted breast density and classified into 3 types: BI-RADS0, BI-RADS1, and BI-RADS2. Moreover, it also classified the abnormalities from the ROIs extracted from these images into benign and malignant. The study also investigated the impact of training size and image size on prediction of accuracy. It was concluded that higher number of training samples improve the prediction accuracy during testing phase. Moreover, rescaling the image size did not have much effect on prediction accuracy of the method. The results show good agreement with manual scores done by expert radiologists.

Summary of the aforementioned methods is presented in Table 2 along with performance metrics for each method and the datasets used in these studies.

**Table 2:** Summary of convolutional neural network–based methods for breast density estimation.

Author	Method	Dataset/number	Task	Performance metric/s (value/s)	Code availability
Mohamed et al [70]	CNN^a^ (AlexNet; transfer learning)	Private, University of Pittsburgh/200,00 DM^b^ (multiview)	Breast density estimation	AUC^c^ (0.9882)	—^d^
Ahn et al [71]	CNN (transfer learning)	Private, Seoul University Hospital/397 DM (multiview)	Breast density estimation	Correlation coefficient (0.96)	—
Xu et al [73]	CNN (scratch based)	Public, INbreast dataset/410 DM (multiview)	Breast density estimation	Accuracy (92.63%)	—
Wu et al [72]	CNN (transfer learning)	Private, New York University School of Medicine/201,179 cases (multiview)	Breast density estimation	Mean AUC (0.934)	[77]
Kallenberg et al [74]	Conventional sparse autoencoder, ie, CNN+stacked autoencoder	Private, Dutch Breast Cancer Screening Program and Mayo Mammography, Minnesota/493+668 images (multiview)	Breast density estimation and risk scoring	Mammographic texture (0.91) and AUC (0.61)	—
Ionescu et al [75]	CNN	Private dataset/67,520 DM (multiview)	Breast density estimation and risk scoring	Average match concordance index of (0.6)	—
Geras et al [76]	Multiview deep neural network	Private, New York University/886,000 image (multiview)	Breast density estimation and risk score	Mean AUC (0.735)	—

^a^CNN: convolutional neural network.

^b^DM: digital mammogram.

^c^AUC: area under the curve.

^d^Not available.

### Convolutional Neural Network for Breast Mass Detection

The automatic detection of masses at an early stage in DMs is still a hot topic of research. DL has significantly overcome the shortcomings of conventional methods by learning the object features. The learning curves of the DL methods have enabled to highlight the most relevant ROIs in DMs. In this section, we present the recent CNN-based methods for the detection of masses in mammograms using transfer learning techniques and scratch-based end-to-end training.

To improve the diagnostic accuracy of the breast CAD system, Dhungel et al [78] introduced a CAD system with minimal user intervention for breast mass detection, segmentation, and classification of the masses. The mass detection is done by cascade DL and random forest model for possible suspected regions that are refined by Bayesian optimization technique. The deep classifier is pretrained with regression analysis and handcrafted features, and the network in fine-tuned bases of ground truths for breast mass classification data, in particular, INbreast dataset, was used for experimentation. Although the method achieved significant results, one of the limitations of this method is that it requires fine-tuning at 2 stages. In addition, it was tested on limited images.

In another study, Dhungel et al [79] proposed a hybrid method for mass segmentation. The proposed conditional random field (CRF) model comprised several potential functions and a DL module for segmentation of masses in mammographic images. The method used tree reweighted (TRW) belief propagation method as a learning mechanism to reduce the lesion segmentation errors and provide optimal results. The study was performed on 2 multiview datasets (ie, INbreast and Digital Database for Screening Mammography [DDSM] datasets). Their results demonstrated that the DL module could improve the classification accuracies when combined with TRW.

Zhu et al [80] proposed a deep structural network with end-to-end learning for the segmentation of masses in DMs. The multistage deep network used a fully convolutional network (FCN) to model a potential function combined with a CRF to perform structured learning. FCN+CRF was used to obtain the empirical estimation of ROIs using the position prior information. To improve the predicted mass estimates, an adversarial training was introduced, which helped to eliminate the overfitting of mass regions with a smaller size in the mammogram dataset. The proposed multistage end-to-end network was evaluated on publicly available datasets (ie, INbreast and DDSM). The results demonstrate the effectiveness of that method.

In another study, Wang et al [81] presented a semiautomated early detection approach using DL to discriminate the microcalcifications and masses in breast cancer dataset. The method aimed to detect the microcalcifications that can be used as an indicator of early breast cancer [82,83]. The DL architecture consisted of stacked autoencoders (SAE) that stack multiple autoencoders, hierarchically. The deep SAE model used layer-wise greedy search training to extract the low-level semantic features of microcalcifications. The method had 2 scenarios: (1) having microcalcification and (2) microcalcifications and masses together to train and test the SAE model. Their method achieved good discriminative accuracy for identifying calcifications using SVM classifier.

**Figure 6:**
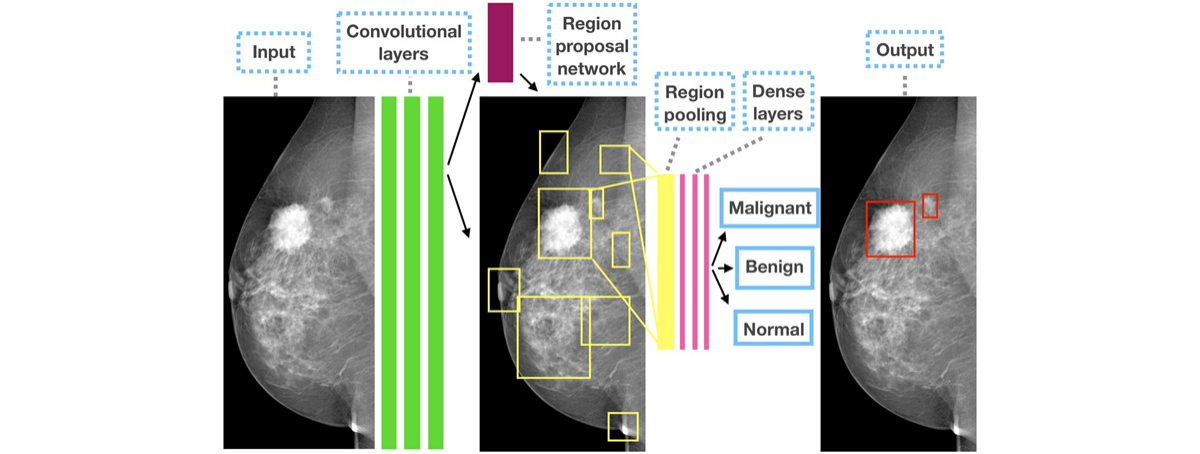
Sample results from the study by Ribli et al for mass detection and classification.

Riddli et al [84] used transfer learning to implement the Faster R-CNN model for the detection of mammographic lesions and classify these lesions into benign and malignant pathology as can be seen in Figure 6 (adapted from [84]). The region proposal network in the Faster R-CNN generated possible suspected regions, which were refined by fine-tuning the hyperparameters. The method achieved significant classification results on the public INbreast database. However, one of the major limitations of this study is that it was tested on a small-scale pixel-level annotated data for detection, whereas the classification task was evaluated on a larger screening dataset.

**Figure 7:**
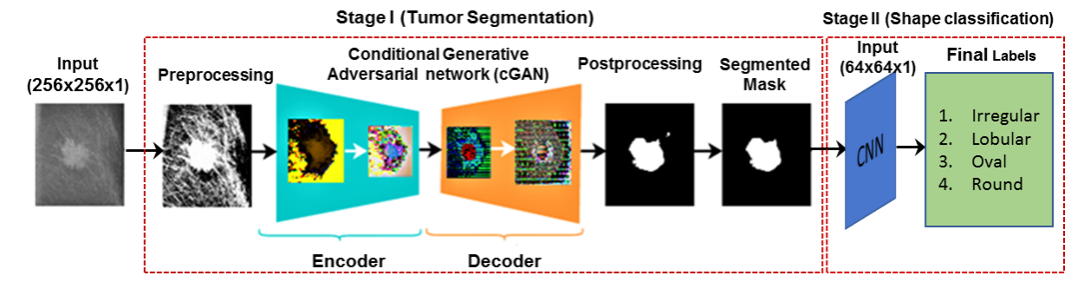
An overview of conditional generative adversarial network adapted from the study by Singh et al for mass segmentation and shape classification. CNN: convolutional neural network.

Singh et al [85] presented a conditional generative adversarial network (cGAN) to segment mammographic masses from a ROI. The generative model learns the lesion representations to create binary masks. Although the adversarial network learns features that discriminate the real masses from the generated binary masks, the key advantage of their proposed cGAN is that it can work well for small sample dataset. The results of their method showed high similarity coefficient value and intersection over union of predicted masses with ground truths. Moreover, the method also classified the detected masses into 4 types (ie, irregular, lobular, oval, and round using CNN) as shown in Figure 7 (adapted from [85]).

Some researchers used image features for lesion detection and classification. One such study by Agarwal and Carson [86] predicted the semantic features such as the type of lesion and pathology in mammograms using the deep CNN. The motivation of the study was to propose a method that could automatically detect lesion and its pathology (ie, calcification or mass either benign or malignant). A scratch-based CNN was trained on DDSM dataset that contained mass as well as calcification cases. The method showed significant results in recognizing the semantic characteristics that can assist the radiologists in clinical decision support task.

Gao et al [87] presented a shallow-deep CNN (SD-CNN) for lesion detection and classification for contrast-enhanced DMs (CEDM). A 4-layered shallow-deep CNN was used to extract the visualization mappings of the convolutional layer in the CEDM images and combine them with low-energy (LE) images. This virtual enhancement improved the quality of LE images. ResNet was applied to these virtual combined images to extract the features to classify benign and normal cases. Using the SD-CNN on the CEDM images resulted in a significant improvement in classification accuracy compared with DMs.

Hagos et al [88] presented a multiview CNN to detect breast masses in symmetrical images. The method used CNN architecture with multiple patches as input to learn the symmetrical differences in the masses. Using the gradient orientation features and local lines on the images, the likelihood of pixels was used to determine the patch as mass or nonmass. They used the AUC and competition performance metric as performance measures for the proposed method against the baseline nonsymmetrical methods.

Later, Tuwen et al [89] proposed a multiview breast mass detection system based on DNN. The 2-step method first detected the suspicious regions in multiview data and then reduced FP through neural learning and affirmed the mass regions. The second major module consists of using transfer learning to train images with Fast R-CNN and mask R-CNN, with 3 different variants of ResNet (ie, ResNet-101, ResNeXt-101, and ResNeXt-152) as backend. The 3 networks were trained on full images to capture enough context information to discriminate soft lesion tissues. Data augmentation was also applied to enrich the dataset.

Jung et al [90] proposed a single-stage masses detection model using the RetinaNet model. RetinaNet is a 1-stage object detection method that can overcome the class imbalance problem and perform better than 2-stage methods. The focal loss function of the model allowed the RetinaNet to focus on the complex sample and detect objects. The mammogram RetinaNet was tested on 2 DM datasets, that is, INbreast and an in-house dataset GURO. Moreover, data augmentation was also used to enrich the database. Using the transfer learning approach, the mass patches from each image were trained using random weight initialization and a different combination. With 6 different experimental settings, the RetinaNet achieved significant detection accuracy compared with other state-of-the-art methods.

Shen et al [91] presented a deep architecture with end-to-end learning to detect and classify the mass regions in the whole digital breast image. The method was trained on the whole mammogram image by using a patch classifier to initiate weights of full image in an end-to-end fashion. The patch classifier uses existing VGG and ResNet architecture for classification. Different combinations of patch sets and hyperparameters were trained to find the optimal combination on whole breast images from the DDSM and INbreast datasets.

We summarize the lesion detection and classification methods in details in Table 3 and illustrate the datasets used, tasks, performance metrics, and code availability.

**Table 3:** Summary of convolutional neural network–based methods for breast mass detection.

Author	Method	Dataset/number	Task	Performance metric/s (value/s)	Code availability
Dhungel et al [78]	Hybrid CNN^a^+level set	Public, INbreast dataset/410 images (multiview)	Mass detection, classification of benign, and malignant	Accuracy (0.9) and sensitivity (0.98)	—^b^
Dhungel et al [79]	CRF^c^+CNN	Public, INbreast and DDSM^d^/116 and 158 images (multiview)	Lesion detection and segmentation	Dice score (0.89)	—
Zhu et al [80]	Fully convolutional network+ CRF	Public, INbreast and DDSM/116 and 158 images (multiview)	Lesion segmentation	Dice score (0.97)	[92]
Wang et al [81]	Stacked autoencoder (transfer learning)	Private, Sun Yat-Sen University/1000 Digital mammogram	Detection and classification of calcifications and masses	Accuracy (0.87)	—
Riddli et al [84]	Faster R-CNN (transfer learning)	Public, DDSM (2620), INbreast (115), and private dataset by Semmelweis University Budapest/847 images	Detection and classification	AUC^e^ (0.95)	Semmelweis dataset: [93]; Code: [94]
Singh et al [85]	Conditional generative adversarial network and CNN	Public and private, DDSM and Reus Hospital Spain dataset/567+194 images	Lesion segmentation and shape classification	Dice score (0.94) and Jaccard Index (0.89)	—
Agarwal and Carson [86]	CNN (scratch based)	Public, DDSM/8750 images (multiview)	Classification of mass and calcifications	Accuracy (0.90)	—
Gao et al [87]	Shallow-deep convolutional neural network, ie, 4 layers CNN+ResNet	Private, Mayo Clinic Arizona (49 subjects) and public, INbreast dataset (89 subjects) (multiview)	Lesion detection and classification	Accuracy (0.9) and AUC (0.92)	—
Hagos et al [88]	Multi-input CNN	Private (General Electric, Hologic, Siemens) dataset/28,294 images/(multiview)	Lesion detection and classification	AUC (0.93) and CPM (0.733)	—
Tuwen et al [89]	Fast R-CNN and Mask R-CNN with ResNet variants as backbone	Private (General Electric, Hologic, Siemens) dataset/23,405 images (multiview)	Lesion detection and classification	Sensitivity (0.97) with 3.56 FP^f^ per image	—
Jung et al [90]	RetinaNet model	Public and private, INbreast and GURO dataset by Korea University Guro Hospital/410+222 images (multiview)	Mass detection and classification	Accuracy (0.98) with 1.3 FP per image	[95]
Shen et al [91]	CNN end-to-end (transfer learning through visual geometry group 16 and ResNet)	Public, DDSM and INbreast/2584 +410 (multiview)	Classification of masses	AUC (0.96)	[96]

^a^CNN: convolutional neural network.

^b^Not available.

^c^CRF: conditional random field.

^d^DDSM: Digital Database for Screening Mammography.

^e^AUC: area under the curve.

^f^FP: false positive.

### Convolutional Neural Network features for Mass Classification

DL algorithms have shown significant improvements in breast cancer detection and classification problem over the past decade. The deep contextual and texture features allow the classifiers to discriminate between normal and abnormal lesions with varying shapes, size, and orientations. This not only improved the diagnostic capabilities of CAD system but also provided robust solutions for clinical practices.

Levy and Jain [97] demonstrated the usefulness of DL as a classification tool to discriminate the benign and malignant cancerous regions. The authors used a transfer learning approach to implement 2 architectures: AlexNet and GoogleNet. Data augmentation is used to increase the number of samples and alleviate overfitting issues. The results showed the significance of DL features in the classification of 2 classes.

Recently, Samala et al [98] presented mass classification method for digital breast tomosynthesis (DBT) using multistage fine-tuned CNN. The method used multistage transfer learning approach using different layer variation and selecting the optimal combination. Initially, the CNN tuned on ImageNet dataset was directly implemented on DBT data, and results were recorded in the multistage CNN that was fine-tuned on DBT dataset. The classification layers of CNN were used with different freeze pattern to extract the best combination that produces the highest accuracy. A total of 6 different combinations of transfer networks with varying freeze pattern for convolutional layers were tested. The multistage transfer learning significantly improved the results with least variations compared with single-stage learning.

Jadoon et al [99] presented a hybrid methodology for breast cancer classification by combining CNN with wavelet and curvelet transform. This model targeted a 3-class classification study (ie, normal, malignant, and benign cases). In this study, 2 methods, namely, CNN-discrete wavelet (CNN-DW) and CNN-curvelet transform (CNN-CT) were used. Features from wavelet and curvelet transform were fused with features obtained from the CNN. Data augmentation was used to enrich the dataset and avoid overfitting of features at the classification stage. Features from CNN-DW and CNN-CT were extracted at 4-level sub-band decompositions separately using the dense scale-invariant features at each sub-band level. The obtained features were presented as input to train a CNN with SoftMax and SVM layer for the classification of normal, benign, and malignant cases.

In a similar study, Huynh et al [100] also used transfer learning and CNN as tools to classify the tumors in breast cancer. The authors proposed an ensemble method that used both CNN and handcrafted features (eg, statistical and morphological features). The features from each method were combined to obtain the ensemble feature matrix. SVM classifier was used with 5-fold cross-validations. Performance of individual methods was compared with the ensemble method using 219 breast lesions. Their results showed that the ensemble could produce better results compared with fine-tuned CNN and analytical feature extractor.

Domingues and Cardoso [101] used an autoencoder to classify the mass versus not mass in the INbreast dataset. The classifier architecture included 1025-500-500-2000-2 layers with the same number of layers for the decoder as well. Except for the last 2 linear layers, all other layers were logistic. The method produced significant results. Moreover, it was also observed that increasing the depth of the network by adding more layers can also improve the detection and classification rates. The authors tested the performance of DL method against 5 classifiers (ie, KNN, decision trees, LDA, Naive Bayes, and SVM).

**Figure 8:**
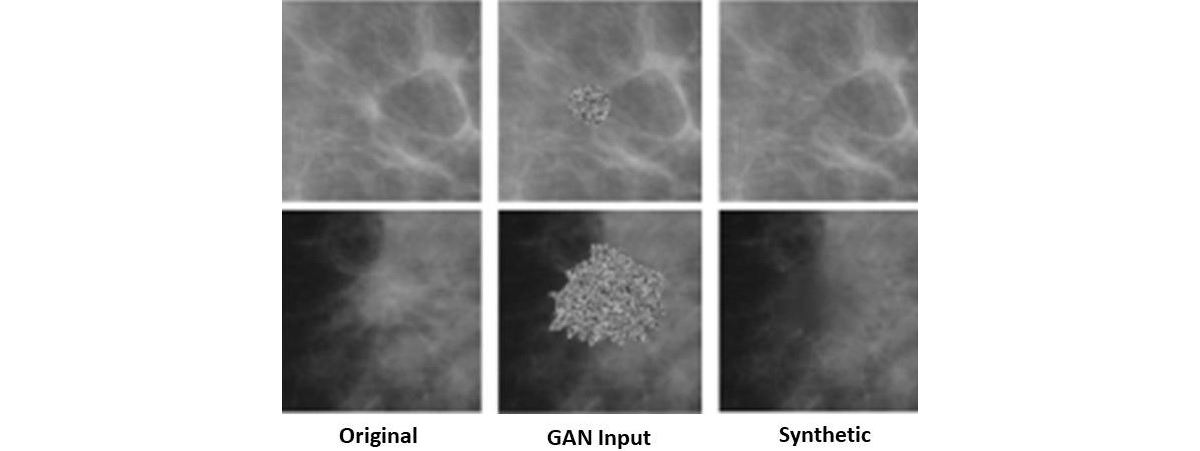
Sample results from the study by Wu et al for synthetic generation of data using conditional generative adversarial network. GAN: generative adversarial network.

Wu et al [102] presented a DL approach to address the class imbalance and limited data issues for breast cancer classification. The approach used the infilling approach to generate synthetic mammogram patches using cGAN network. In the first step, the multiscale generator was trained to create synthetic patches in the target image using GAN. The generator used a cascading refinement to generate the multiscale features to ensure stability at high resolution. Figure 8 shows the synthetic images generated by cGAN. The cGAN was restricted to infill only lesion either mass or calcifications. The quality of generated images was experimentally evaluated by training a ResNet-50 classifier. The classification performance of cGAN augmented, and traditional augmentation methods were also compared. The results showed that synthetic augmentation improves classification.

Sarah et al [103] addressed the issue of reducing the recall rates in breast cancer diagnosis. The higher number of FP results in higher recalls, which leads to unnecessary biopsies and increased cost for the patients. In this study, a DL method to reduce the recall rates was proposed. A deep CNN, namely, AlexNet, was implemented. A total of 6 different scenarios of mammogram classification were investigated. CNN was able to discriminate and classify these 6 categories very efficiently. Moreover, it could also be inferred that some features in recalled benign images classify them reexamined and to be recalled instead of classifying them as negative (normal) cases.

Lately, Wang et al [104] presented a hybrid DL method for multiview breast mass diagnosis. The framework exploited the contextual information from the multiview data (ie, CC and MLO) using CNN features and attention mechanism. The proposed multiview DNN aimed to help medical experts for the classification of breast cancer lesion. The method comprised 4 steps, and mass cropping and extraction of clinical features were done from the multiview patches. The recurrent neural network, in particular, long short-term memory, was used to extract the label co-occurrence dependency of multiview information for the classification of mass regions into benign and malignant cases using the clinical and CNN features as input.

In another study, Shams et al [105] proposed a GAN-based mammogram classification method—Deep GeneRAtive Multitask (DiaGRAM) network to deal with data scarcity and limited availability of annotated data. The DiaGRAM effectively uses an end-to-end multitask learning to improve diagnostic performance on limited number of datasets.

Gastitouni et al [106] presented an ensemble method for breast pectoral parenchymal classification. The texture feature maps extracted from lattice-based techniques are fed as input separately to a multichannel CNN. The meta-features from the CNN predicted the risk score associated with breast parenchyma. The hybrid method showed better performance compared with individual texture features and CNN, respectively.

Dhungel et al [107] introduced a multiview ensemble deep ResNet (mResNet) for classification of malignant and benign tumors. Their ensemble network comprised deep ResNet capable to tackle 6 input images, with different views, that is, CC and MLO. The mResNet can automatically produce binary maps of the lesions. The final output of the mResNet are concatenated to obtain a fully connected layer that can classify the lesions into malignant or benign class.

Generally, DL methods have significantly improved the performance of breast cancer detection, classification, and segmentation. We summarize these methods in details in Table 4 and illustrate the datasets used, tasks, performance metrics, and code availability.

**Table 4:** Summary of convolutional neural network–based methods for breast mass classification.

Author	Method	Dataset/number	Task	Performance metric/s (value/s)	Code availability
Levy and Jain [97]	AlexNet and GoogleNet (transfer learning)	Public, DDSM^a^ dataset/1820 images (multiview)	Breast mass classification	Accuracy (0.924), precision (0.924), and recall (0.934)	—^b^
Samala et al [98]	Multistage fine-tuned CNN^c^ (transfer learning)	Private+public, University of Michigan and DDSM/4039 ROIs^d^ (multiview)	Classification performance on varying sample sizes	AUC^e^ (0.91)	[108]
Jadoon et al [99]	CNN- Discrete wavelet and CNN-curvelet transform	Public, image retrieval in medical applications dataset/2796 ROI patches	Classification	Accuracy (81.83 and 83.74) and receiver operating characteristic curve (0.831 and 0.836) for both methods	—
Huynh et al [100]	CNN (transfer learning)	Private, University of Chicago/219 images (multiview)	Classification of benign and malignant tumor	AUC (0.86)	—
Domingues and Cardoso [101]	Autoencoder	Public, INbreast/116 ROIs	Classification of mass vs normal	Accuracy (0.99)	[109]
Wu et al [102]	GAN^f^ and ResNet50	Public, DDSM dataset/10,480 images (multiview)	Detection and classification of benign and malignant calcifications and masses	AUC (0.896)	[110]
Sarah et al [103]	CNN (transfer learning)	Public, Full-field digital mammography and DDSM/14,860 images (multiview)	Classification	AUC (0.91)	—
Wang et al [104]	CNN and long short-term memory	Public, Breast Cancer Digital Repository (BCDR-F03)/763 images (multiview)	Classification of breast masses using contextual information	AUC (0.89)	—
Shams et al [105]	CNN and GAN	Public, INbreast and DDSM (multiview)	Classification	AUC (0.925)	—
Gastounioti et al [106]	Texture feature+CNN	Private/106 cases (mediolateral oblique view only)	Classification	AUC (0.9)	—
Dhungel et al [107]	Multi-ResNet	Public, INbreast (multiview)	Classification	AUC (0.8)	—

^a^DDSM: Digital Database for Screening Mammography.

^b^Not available.

^c^CNN: convolutional neural network.

^d^ROIs: region of interest.

^e^AUC: area under the curve.

^f^GAN: generative adversarial network.

## Discussion

### Principal Findings

From Tables 2, 3, and 4, it can be noted that significant works have been done on breast cancer diagnosis. The review of breast diagnosis methods shows that DL has helped to improve the diagnostic performance of the breast CAD system, but still challenges remain for clinical applicability of such methods, and more research is needed. The presented literature aims to help in building a CAD system that is robust, computationally efficient to assist the clinicians in the diagnosis of breast cancer at early stages. One main problem related to mammograms is the heterogeneity of breast tissues; that is, the images acquired at CC and MLO view may not show with different densities. Some researchers use breast density estimation scores as the initial biomarker for the presence of cancer. However, the analysis shows that these methods can be confined to a particular type of breast density and cannot be generalized for the whole population. Others use DL in a hybrid approach and a semi supervised manner to extract significant semantic and contextual information to detect and classify the breast lesions.

On the other hand, many attempts have been made to reduce human intervention and produce fully automatic CAD system, which is a very challenging task. In fact, all methods in literature require annotated images (ground truth) to validate their findings during the training and testing stages. Thus, acquisition of labeled mammograms with image-level and pixel-level annotations is one of the obstacles in designing robust DL methods. The main issue is not only the availability of data but also annotations by expert radiologist, which is time consuming, subjective, and expensive.

It is noted from the literature that the automated DL method requires extensive experimentation, computational power, and preprocessing of data, which make it inefficient to be used in real time. Moreover, finding the optimal parameters in DL networks is also one of the major challenges in building a CAD system for clinical use. However, this issue can be resolved if sufficient training is provided to clinicians, and CAD systems are made more user friendly. It is also noted that the semisupervised approaches have shown good performance on the public and private datasets for breast cancer diagnosis.

From the analysis of methods mentioned in Tables 2, 3, and 4, it can be noted that most methods mentioned previously adapt the augmentation strategies to enrich the dataset. All these techniques only use geometric transformations to create rotated and scale version of existing samples without adding any morphological variations in the lesions. Thus, enrichment of data with more samples is only limited to affine transformations and cannot fully resolve the overfitting problem.

Developing DL models that can learn from limited data is still an open research area not only in breast cancer diagnosis but also for other medical image analysis applications. Moreover, developing data augmentation techniques that can create morphological variations in augmented samples, while also preserving the lesion characteristic, are needed. One of the solutions to address these problems is to explore the capabilities of GANs as successfully demonstrated in studies by Singh et al [85] and Wu et al [102]. Techniques such as these will not only tackle the insufficiency issue of data but will also provide a viable solution to class imbalance problem, which is also an important research area.

Apart from the development of automatic DL techniques, there are other associated challenges to the medical imaging research community. First, it is very challenging to secure funding for construction of a medical dataset. Also, finding an expert for annotation and the cost of annotation itself is very high. Second, privacy and copyright issues make the medical image difficult to share compared with natural images datasets. Finally, because of the complex anatomy of human organs, a variety of dataset is required using different imaging modalities. Despite these challenges, there has been a significant increase in the number of public datasets. Organizing a grand challenge is one of the good practices devised to share and enrich the datasets. The participants are provided with a certain number of tasks on a particular dataset, and the technique with best results is announced as a winner. Moreover, different research centers join hands in research collaborations as well as common data sharing platforms.

### Conclusions

From the aforementioned discussions, we can see that both supervised and unsupervised DL methods are used by the image analysis community, but the majority of the work uses the semi supervised approach. The presented literature aims to help in building a CAD system that is robust and computationally efficient to assist the clinicians in the diagnosis of breast cancer at early stages. As DL requires a sufficient amount of annotated data for training, most of the researchers use a combination of public and private data followed by data augmentation techniques to overcome the data scarcity issue. These approaches have provided a feasible solution to the problem of scarcity of data and overfitting.

## Appendix

Multimedia Appendix 1 Commonly used metrics for performance evaluation in breast cancer diagnosis.

## Abbreviations

BI-RADS: Breast Imaging Reporting and Data System
CAD: computer-aided diagnosis
CC: craniocaudal
CEDM: contrast-enhanced digital mammograms
cGAN: conditional generative adversarial network
CNN: convolutional neural network
CRF: conditional random field
CT: curvelet transform
DBT: digital breast tomosynthesis
DiaGRAM: Deep Generative Multitask
DL: deep learning
DM: digital mammogram
DW: discrete wavelet
FCN: fully convolutional network
FP: false positive
IARC: International Agency for Cancer Research
KNN: k-nearest neighbor
LDA: linear discriminant analysis
LE: low energy
ML: machine learning
MLO: mediolateral oblique
PM: pectoral muscle
ReLU: rectified linear unit
ResNet: residual network
ROI: region of interest
SAE: stacked autoencoder
SD-CNN: shallow-deep convolutional neural network
SVM: support vector machine
TRW: tree reweighted
VGG: visual geometry group
WHO: World Health Organization

## References

[1] (2018). American Cancer Society.

[2] Blakely T, Shaw C, Atkinson J, Cunningham R, Sarfati D (2011). Social inequalities or inequities in cancer incidence? Repeated census-cancer cohort studies, New Zealand 1981-1986 to 2001-2004. Cancer Causes Control.

[3] Smigal C, Jemal A, Ward E, Cokkinides V, Smith R, Howe HL, Thun M (2006). Trends in breast cancer by race and ethnicity: update 2006. CA Cancer J Clin.

[4] (2012). Guide to Mammography And Other Breast Imaging Procedures.

[5] Ponraj DN, Jenifer ME, Poongodi DP, Manoharan JS (2011). A survey on the preprocessing techniques of mammogram for the detection of breast cancer. J Emerg Trends Comput Inf Sci.

[6] Rangayyan RM, Ayres FJ, Leo Desautels JE (2007). A review of computer-aided diagnosis of breast cancer: toward the detection of subtle signs. J Franklin Inst.

[7] Ganesan K, Acharya UR, Chua KC, Min LC, Abraham KT (2013). Pectoral muscle segmentation: a review. Comput Methods Programs Biomed.

[8] Ge M, Mainprize JG, Mawdsley GE, Yaffe MJ (2014). Segmenting pectoralis muscle on digital mammograms by a Markov random field-maximum a posteriori model. J Med Imaging (Bellingham).

[9] Ali MA, Czene K, Eriksson L, Hall P, Humphreys K (2017). Breast tissue organisation and its association with breast cancer risk. Breast Cancer Res.

[10] Oliver A, Lladó X, Torrent A, Martí J (2014). One-Shot Segmentation of Breast, Pectoral Muscle, and Background in Digitised Mammograms. Proceedings of the International Conference on Image Processing.

[11] Bora VB, Kothari AG, Keskar AG (2016). Robust automatic pectoral muscle segmentation from mammograms using texture gradient and euclidean distance regression. J Digit Imaging.

[12] Tourassi GD, Vargas-Voracek R, Catarious Jr DM, Floyd Jr CE (2003). Computer-assisted detection of mammographic masses: a template matching scheme based on mutual information. Med Phys.

[13] Rampun A, Morrow PJ, Scotney BW, Winder J (2017). Fully automated breast boundary and pectoral muscle segmentation in mammograms. Artif Intell Med.

[14] Eltoukhy MM, Faye I (2013). An Adaptive Threshold Method for Mass Detection in Mammographic Images. Proceedings of the International Conference on Signal and Image Processing Applications.

[15] Gardezi SJ, Faye I, Sanchez BJ, Kamel N, Hussain M (2017). Mammogram classification using dynamic time warping. Multimed Tools Appl.

[16] Shih FY (2010). Image Processing and Pattern Recognition: Fundamentals and Techniques.

[17] Biltawi M, Al-Najdawi N, Tedmori S (2012). Mammogram Enhancement and Segmentation Methods: Classification, Analysis, and Evaluation. Proceedings of the 13th International Arab Conference on Information Technology.

[18] de Oliveira HC, Mencattini A, Casti P, Martinelli E, di Natale C, Catani JH, de Barros N, Melo CF, Gonzaga A, Vieira MA (2018). Reduction of False-Positives in a CAD Scheme for Automated Detection of Architectural Distortion in Digital Mammography. Proceeding of the Conference on Computer-Aided Diagnosis.

[19] Liu X, Zeng Z (2015). A new automatic mass detection method for breast cancer with false positive reduction. Neurocomputing.

[20] Jen CC, Yu SS (2015). Automatic detection of abnormal mammograms in mammographic images. Expert Syst Appl.

[21] Ayer T, Chen Q, Burnside ES (2013). Artificial neural networks in mammography interpretation and diagnostic decision making. Comput Math Methods Med.

[22] Magna G, Casti P, Jayaraman SV, Salmeri M, Mencattini A, Martinelli E, Natale CD (2016). Identification of mammography anomalies for breast cancer detection by an ensemble of classification models based on artificial immune system. Knowl Based Syst.

[23] Wang H, Zheng B, Yoon SW, Ko HS (2018). A support vector machine-based ensemble algorithm for breast cancer diagnosis. Eur J Oper Res.

[24] Sert E, Ertekin S, Halici U (2017). Ensemble of Convolutional Neural Networks for Classification of Breast Microcalcification From Mammograms. Proceedings of the 39th Annual International Conference of the IEEE Engineering in Medicine and Biology Society.

[25] Cheng H, Shi X, Min R, Hu L, Cai X, Du H (2006). Approaches for automated detection and classification of masses in mammograms. Pattern Recognit.

[26] Hasan H, Tahir NM (2010). Feature Selection of Breast Cancer Based on Principal Component Analysis. Proceedings of the 6th International Colloquium on Signal Processing & Its Applications.

[27] Chan HP, Wei D, Helvie MA, Sahiner B, Adler DD, Goodsitt MM, Petrick N (1995). Computer-aided classification of mammographic masses and normal tissue: linear discriminant analysis in texture feature space. Phys Med Biol.

[28] Jin X, Xu A, Bie R, Guo P (2006). Machine Learning Techniques and Chi-square Feature Selection for Cancer Classification Using SAGE Gene Expression Profiles. Proceedings of the 2006 International Conference on Data Mining for Biomedical Applications.

[29] Salama GI, Abdelhalim MB, Zeid MA (2012). Breast cancer diagnosis on three different datasets using multi-classifiers. Int J Comput Sci Inf Technol.

[30] Saghapour E, Kermani S, Sehhati M (2017). A novel feature ranking method for prediction of cancer stages using proteomics data. PLoS One.

[31] Eltoukhy MM, Gardezi SJ, Faye I (2014). A Method to Reduce Curvelet Coefficients for Mammogram Classification. Proceedings of the Region 10 Symposium.

[32] Singh B, Jain V, Singh S (2014). Mammogram mass classification using support vector machine with texture, shape features and hierarchical centroid method. J Med Imaging & Health Infor.

[33] Sonar P, Bhosle U, Choudhury C (2017). Mammography Classification Using Modified Hybrid SVM-KNN. Proceedings of the International Conference on Signal Processing and Communication.

[34] Gardezi SJ, Faye I, Eltoukhy MM (2014). Analysis of Mammogram Images Based on Texture Features of Curvelet Sub-Bands. Proceedings of the 5th International Conference on Graphic and Image Processing.

[35] Pratiwi M, Harefa J, Nanda S, Alexander (2015). Mammograms classification using gray-level co-occurrence matrix and radial basis function neural network. Procedia Comput Sci.

[36] Pal NR, Bhowmick B, Patel SK, Pal S, Das J (2008). A multi-stage neural network aided system for detection of microcalcifications in digitized mammograms. Neurocomputing.

[37] Wu X, Kumar V, Ross QJ, Ghosh JR, Yang Q, Motoda H, McLachlan GJ, Ng A, Liu B, Yu PS, Zhou Z, Steinbach M, Hand DJ, Steinberg D (2007). Top 10 algorithms in data mining. Knowl Inf Syst.

[38] Tan PN, Steinbach M, Kumar V (2006). Introduction to Data Mining.

[39] Sumbaly R, Vishnusri N, Jeyalatha S (2014). Diagnosis of breast cancer using decision tree data mining technique. Int J Comput Appl.

[40] Landwehr N, Hall M, Frank E (2005). Logistic model trees. Mach Learn.

[41] Ramirez-Villegas JF, Ramirez-Moreno DF (2012). Wavelet packet energy, Tsallis entropy and statistical parameterization for support vector-based and neural-based classification of mammographic regions. Neurocomputing.

[42] Wajid SK, Hussain A (2015). Local energy-based shape histogram feature extraction technique for breast cancer diagnosis. Expert Syst Appl.

[43] Zhang X, Homma N, Goto S, Kawasumi Y, Ishibashi T, Abe M, Sugita N, Yoshizawa M (2013). A hybrid image filtering method for computer-aided detection of microcalcification clusters in mammograms. J Med Eng.

[44] Abbas Q, Celebi ME, García If (2013). Breast mass segmentation using region-based and edge-based methods in a 4-stage multiscale system. Biomed Signal Process Control.

[45] Jiang M, Zhang S, Li H, Metaxas DN (2015). Computer-aided diagnosis of mammographic masses using scalable image retrieval. IEEE Trans Biomed Eng.

[46] Michaelson J, Satija S, Moore R, Weber G, Halpern E, Garland A, Kopans DB, Hughes K (2003). Estimates of the sizes at which breast cancers become detectable on mammographic and clinical grounds. J Womens Health.

[47] Krizhevsky A, Sutskever I, Hinton GE (2017). ImageNet classification with deep convolutional neural networks. Commun ACM.

[48] Young T, Hazarika D, Poria S, Cambria E (2018). Recent trends in deep learning based natural language processing. IEEE Comput Intell Mag.

[49] Schuurmans D, Zinkevich MA (2016). Deep Learning Games. Proceedings of the Advances in Neural Information Processing Systems.

[50] Yang X, Zeng Z, Yeo SY, Tan C, Tey HL, Su Y (2017). Cornell University.

[51] Esteva A, Kuprel B, Novoa RA, Ko J, Swetter SM, Blau HM, Thrun S (2017). Dermatologist-level classification of skin cancer with deep neural networks. Nature.

[52] Havaei M, Davy A, Warde-Farley D, Biard A, Courville A, Bengio Y, Pal C, Jodoin PM, Larochelle H (2017). Brain tumor segmentation with deep neural networks. Med Image Anal.

[53] Lecun Y, Bottou L, Bengio Y, Haffner P (1998). Gradient-Based Learning Applied to Document Recognition. Proceedings of the IEEE.

[54] Simonyan K, Zisserman A (2014). Cornell University.

[55] Szegedy C, Liu W, Jia Y, Sermanet P, Reed S, Anguelov D (2015). Going Deeper With Convolutions. Proceedings of the Conference on Computer Vision and Pattern Recognition.

[56] He K, Zhang X, Ren S, Sun J (2016). Identity Mappings in Deep Residual Networks. Proceedings of the Conference on Computer Vision.

[57] He K, Zhang X, Ren S, Sun J (2016). Deep Residual Learning for Image Recognition. Proceedings of the Conference on Computer Vision and Pattern Recognition.

[58] Tajbakhsh N, Shin JY, Gurudu SR, Hurst RT, Kendall CB, Gotway MB, Jianming L (2016). Convolutional neural networks for medical image analysis: full training or fine tuning?. IEEE Trans Med Imaging.

[59] Suk HI, Lee SW, Shen D, Alzheimer's Disease Neuroimaging Initiative (2014). Hierarchical feature representation and multimodal fusion with deep learning for AD/MCI diagnosis. Neuroimage.

[60] Cheng JZ, Ni D, Chou YH, Qin J, Tiu CM, Chang YC, Huang C, Shen D, Chen C (2016). Computer-aided diagnosis with deep learning architecture: applications to breast lesions in US images and pulmonary nodules in CT scans. Sci Rep.

[61] Ting FF, Tan YJ, Sim KS (2019). Convolutional neural network improvement for breast cancer classification. Expert Syst Appl.

[62] Setio AA, Ciompi F, Litjens G, Gerke P, Jacobs C, van Riel SJ, Wille MM, Naqibullah M, Sanchez CI, van Ginneken B (2016). Pulmonary nodule detection in CT images: false positive reduction using multi-view convolutional networks. IEEE Trans Med Imaging.

[63] Shin HC, Roth HR, Gao M, Lu L, Xu Z, Nogues I, Yao J, Mollura D, Summers RM (2016). Deep convolutional neural networks for computer-aided detection: CNN architectures, dataset characteristics and transfer learning. IEEE Trans Med Imaging.

[64] Shie CK, Chuang CH, Chou CN, Wu MH, Chang EY (2015). Transfer Representation Learning for Medical Image Analysis. Proceedings of the Engineering in Medicine and Biology Society.

[65] Qi D, Hao C, Lequan Y, Lei Z, Jing Q, Defeng W, Mok VC, Lin S, Pheng-Ann H (2016). Automatic detection of cerebral microbleeds from MR images via 3D convolutional neural networks. IEEE Trans Med Imaging.

[66] Lehman CD, Yala A, Schuster T, Dontchos B, Bahl M, Swanson K, Barzilay R (2019). Mammographic breast density assessment using deep learning: clinical implementation. Radiology.

[67] Sprague BL, Conant EF, Onega T, Garcia MP, Beaber EF, Herschorn SD, Lehman CD, Tosteson AN, Lacson R, Schnall MD, Kontos D, Haas JS, Weaver DL, Barlow WE, PROSPR Consortium (2016). Variation in mammographic breast density assessments among radiologists in clinical practice: a multicenter observational study. Ann Intern Med.

[68] Youk JH, Gweon HM, Son EJ, Kim JA (2016). Automated volumetric breast density measurements in the era of the BI-RADS fifth edition: a comparison with visual assessment. AJR Am J Roentgenol.

[69] Brandt KR, Scott CG, Ma L, Mahmoudzadeh AP, Jensen MR, Whaley DH, Wu FF, Malkov S, Hruska CB, Norman AD, Heine J, Shepherd J, Pankratz VS, Kerlikowske K, Vachon CM (2016). Comparison of clinical and automated breast density measurements: implications for risk prediction and supplemental screening. Radiology.

[70] Mohamed AA, Berg WA, Peng H, Luo Y, Jankowitz RC, Wu S (2018). A deep learning method for classifying mammographic breast density categories. Med Phys.

[71] Ahn CK, Heo C, Jin H, Kim JH (2017). A Novel Deep Learning-Based Approach to High Accuracy Breast Density Estimation in Digital Mammography. Proceedings of the Computer-Aided Diagnosis.

[72] Wu N, Geras KJ, Shen Y, Su J, Kim SG, Kim E (2018). Breast Density Classification With Deep Convolutional Neural Networks. Proceedings of the International Conference on Acoustics, Speech and Signal Processing.

[73] Xu J, Li C, Zhou Y, Mou L, Zheng H, Wang S (2018). Cornell University.

[74] Kallenberg M, Petersen K, Nielsen M, Ng AY, Pengfei D, Igel C, Vachon CM, Holland K, Winkel RR, Karssemeijer N, Lillholm M (2016). Unsupervised deep learning applied to breast density segmentation and mammographic risk scoring. IEEE Trans Med Imaging.

[75] Ionescu GV, Fergie M, Berks M, Harkness EF, Hulleman J, Brentnall AR, Cuzick J, Evans DG, Astley SM (2019). Prediction of reader estimates of mammographic density using convolutional neural networks. J Med Imaging (Bellingham).

[76] Geras KJ, Wolfson S, Shen Y, Kim S, Moy L, Cho K (2017). Cornell University.

[77] GitHub.

[78] Dhungel N, Carneiro G, Bradley A (2017). A deep learning approach for the analysis of masses in mammograms with minimal user intervention. Med Image Anal.

[79] Dhungel N, Carneiro G, Bradley AP (2015). Tree RE-Weighted Belief Propagation Using Deep Learning Potentials for Mass Segmentation From Mammograms. Proceedings of the 12th International Symposium on Biomedical Imaging.

[80] Zhu W, Xiang X, Tran TD, Hager GD, Xie X (2018). Adversarial Deep Structured Nets for Mass Segmentation From Mammograms. Proceedings of the 15th International Symposium on Biomedical Imaging.

[81] Wang J, Yang X, Cai H, Tan W, Jin C, Li L (2016). Discrimination of breast cancer with microcalcifications on mammography by deep learning. Sci Rep.

[82] Fletcher SW, Elmore JG (2003). Clinical practice. Mammographic screening for breast cancer. N Engl J Med.

[83] Yunus M, Ahmed N, Masroor I, Yaqoob J (2004). Mammographic criteria for determining the diagnostic value of microcalcifications in the detection of early breast cancer. J Pak Med Assoc.

[84] Ribli D, Horváth A, Unger Z, Pollner P, Csabai I (2018). Detecting and classifying lesions in mammograms with deep learning. Sci Rep.

[85] Singh VK, Romani S, Rashwan HA, Akram F, Pandey N, Sarker M (2018). Conditional Generative Adversarial and Convolutional Networks for X-ray Breast Mass Segmentation and Shape Classification. Proceedings of the Medical Image Computing and Computer Assisted Intervention.

[86] Agarwal V, Carson C (2015). Stanford University.

[87] Gao F, Wu T, Li J, Zheng B, Ruan L, Shang D, Patel B (2018). SD-CNN: a shallow-deep CNN for improved breast cancer diagnosis. Comput Med Imaging Graph.

[88] Hagos YB, Mérida AG, Teuwen J (2018). Improving Breast Cancer Detection Using Symmetry Information with Deep Learning. Proceedings of the Image Analysis for Moving Organ, Breast, and Thoracic Images.

[89] Teuwen J, van de Leemput S, Gubern-Mérida A, Rodriguez-Ruiz A, Mann R, Bejnordi B (2018). Soft Tissue Lesion Detection in Mammography Using Deep Neural Networks for Object Detection. Proceedings of the 1st Conference on Medical Imaging with Deep Learning.

[90] Jung H, Kim B, Lee I, Yoo M, Lee J, Ham S, Woo O, Kang J (2018). Detection of masses in mammograms using a one-stage object detector based on a deep convolutional neural network. PLoS One.

[91] Shen L, Laurie LM, Joseph HR, Eugene F, Russell B, Weiva S (2017). Cornell University.

[92] GitHub.

[93] [Department of Radiology].

[94] GitHub.

[95] GitHub.

[96] GitHub.

[97] Lévy D, Jain A (2016). Cornell University.

[98] Samala RK, Chan HP, Hadjiiski L, Helvie MA, Richter CD, Cha KH (2019). Breast cancer diagnosis in digital breast tomosynthesis: effects of training sample size on multi-stage transfer learning using deep neural nets. IEEE Trans Med Imaging.

[99] Jadoon MM, Zhang Q, Haq IU, Butt S, Jadoon A (2017). Three-class mammogram classification based on descriptive CNN features. Biomed Res Int.

[100] Huynh BQ, Li H, Giger ML (2016). Digital mammographic tumor classification using transfer learning from deep convolutional neural networks. J Med Imaging (Bellingham).

[101] Domingues I, Cardoso J (2013). Mass Detection on Mammogram Images: A First Assessment of Deep Learning Techniques. Proceedings of the 19th edition of the Portuguese Conference on Pattern Recognition.

[102] Wu E, Wu K, Cox D, Lotter W (2018). Conditional Infilling GANs for Data Augmentation in Mammogram Classification. Proceedings of the Image Analysis for Moving Organ, Breast, and Thoracic Images.

[103] Aboutalib SS, Mohamed AA, Berg WA, Zuley ML, Sumkin JH, Wu S (2018). Deep learning to distinguish recalled but benign mammography images in breast cancer screening. Clin Cancer Res.

[104] Wang H, Feng J, Zhang Z, Su H, Cui L, He H, Liu L (2018). Breast mass classification via deeply integrating the contextual information from multi-view data. Pattern Recognit.

[105] Shams S, Platania R, Zhang J, Kim J, Lee K, Park SJ (2018). Deep Generative Breast Cancer Screening and Diagnosis. Proceedings of the Medical Image Computing and Computer Assisted Intervention.

[106] Gastounioti A, Oustimov A, Hsieh MK, Pantalone L, Conant EF, Kontos D (2018). Using convolutional neural networks for enhanced capture of breast parenchymal complexity patterns associated with breast cancer risk. Acad Radiol.

[107] Dhungel N, Carneiro G, Bradley AP (2017). Fully Automated Classification of Mammograms Using Deep Residual Neural Networks. Proceedings of the 14th International Symposium on Biomedical Imaging.

[108] IEEE Xplore Digital Library.

[109] Department of Computer Science, University of Toronto.

[110] GitHub.

