# Supplementary material for: Breast Cancer Detection and Diagnosis Using Mammographic Data: Systematic Review
Source: J Med Internet Res. 2019 Jul 26;21(7):e14464. doi: 10.2196/14464 (PMC6688437; doi:10.2196/14464)
Supplement: Multimedia Appendix 1 [file jmir_v21i7e14464_app1.pdf]

The most commonly used performance evaluation metric in a CAD system are given as follows:

The accuracy of test is the number of correctly diagnosed cases in a dataset and expressed below:

$$Accuracy = \frac{(TPs)+(TNs)}{(TPs)+(TNs)+(FPs)+(FNs)} \quad (1)$$

The sensitivity is the measure of proportions of positively diagnosed cases that are correctly identified and expressed as:

$$Sensitivity = \frac{(TPs)}{(TPs)+(FNs)} \quad (2)$$

The specificity is the measure of proportions of negatives that are correctly identified and expressed as

$$Specificity = \frac{(TNs)}{(TNs)+(FPs)} \quad (3)$$

where in Equations (1-3)

True positive (TP) = Abnormal cases correctly identified as abnormal.

False positive (FP) = Normal cases identified incorrectly as abnormal.

True negative (TN) = Normal cases correctly identified as normal.

Jaccard Index:

The jaccard index (*J*) measures the similarity of two samples and mathematically is presented as follows:

$$J(I_o, I_c) = \frac{|I_o \cap I_c|}{|I_o \cup I_c|} \quad (4)$$

where I_o is orginal and I_c is the predicted image under study. The value of $J(I_o, I_c)$ lies between $0 \leq J(I_o, I_c) \leq 1$. Higher values of *J* show strong agreement between original and predicted values.

Dice Score:

The Dice score to calculate the similarity of two images is defined as

$$DS(I_o, I_c) = 2 * \frac{|I_o \cap I_c|}{|I_o|+|I_c|} \quad (5)$$

The objects with higher Dice score exhibit higher similarities.
